# OCB-Work-Family Facilitation: Is It Positive for All Attachment Orientations?

**DOI:** 10.3389/fpsyg.2019.02900

**Published:** 2020-01-14

**Authors:** Abira Reizer, Meni Koslowsky, Batel Friedman

**Affiliations:** Department of Behavioral Sciences, Ariel University, Ariel, Israel

**Keywords:** organizational citizenship behavior, work-family facilitation, attachment, avoidance, anxiety, diary assessments

## Abstract

The present study seeks to expand on research concerning the benefits of organizational citizenship behaviors (OCBs) to work-family facilitation (WFF) by integrating the theoretical framework of the attachment personality perspective (Bowlby, 1982). We hypothesized that OCB would enhance WFF for employees having lower levels of avoidance and anxious orientations but reduce WFF for employees with higher levels of avoidance and anxiety orientations. Two studies were conducted to test these hypotheses. Study 1 adopted a cross-sectional design, and Study 2 implemented a diary procedure. In Study 1, employees from a pharmaceutical company completed attachment orientations and WFF questionnaires, whereas their direct supervisors assessed the participants’ OCB. In Study 2, attachment orientations of 108 participants were assessed, with OCB and WFF measures collected over 10 days. Findings from both studies supported our hypotheses relating to avoidance orientations. Performing OCB can enhance WFF, with the effect stronger for employees having lower avoidance orientations. However, findings regarding anxiety orientations were non-significant. A better understanding of the role that attachment orientations play in the OCB – WFF association may facilitate implementing possible interventions that could benefit both the organization and the family.

## Introduction

Previous research has shown that organizations benefit when employees contribute beyond the formal definition of their job requirements, commonly referred to as organizational citizenship behavior (OCB; Organ et al., 2006; Organ, 2018). Researchers tend to explain the positive effects of OCB through the enriching lens (Lam et al., 2016). For example, in their meta-analysis, Podsakoff et al. (2009) indicated that OCB is beneficial, both at the individual and the organizational levels, by simplifying maintenance functions, freeing up resources for productivity, improving service quality, and enhancing performance. At the individual level, performing OCB increases employees’ positive emotions (Glomb et al., 2011) and vigor (Lam et al., 2016). Moreover, through active engagement in OCB, employees may enjoy personal privilege benefits or other personal gains (such as higher status, social ties, or job promotion; for a review, see Bolino and Grant, 2016). In the current study, we examined if and how OCBs that are performed at work can offer additional benefits outside the workplace, such as work-family facilitation (WFF).

Recently, a growing interest in the literature on OCB has suggested that even good things can lead to adverse outcomes and showed that OCB can be a time-consuming activity (Bolino et al., 2012) that distracts the employee from performing his or her own working assignments (Koopman et al., 2016) and increases burnout (Bolino et al., 2018). We suggest that individual differences in personality traits can clarify when OCB performance is beneficial and for whom it is less effective. Specifically, we propose attachment as a personality moderator in the OCB- WFF relationship. Attachment orientations enable understanding of the human capacity to connect with others and develop supportive relationships. These orientations are considered fundamental personality tendencies, offering a theoretical foundation and a well-validated body of empirical evidence in the social and personality fields, as reflected in numerous studies (for a review, see Mikulincer and Shaver, 2017). Indeed, the organizational and management literature investigating the effect of attachment orientations at the workplace has significantly grown during the last decade, particularly over the last 5 years (Yip et al., 2018; Reizer, 2019). Whereas previous research has found attachment orientations to predict various behavioral and organizational outcomes, two extensive reviews of the literature (Harms, 2011; Yip et al., 2018) strongly argued for future researchers to incorporate attachment as a potential moderator of organizational processes.

**FIGURE 1:**
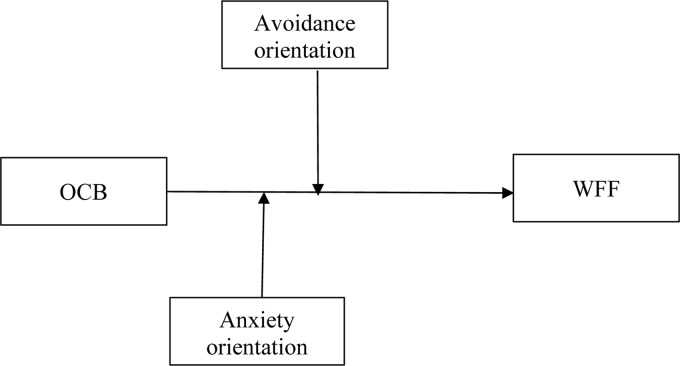
Proposed research model.

Therefore, this study’s objectives are twofold: first, we expand on previous work examining the positive benefits of OCB by empirically investigating the impact of OCB over WFF. Second, in considering attachment personality orientations, we examine whether employees’ subjective experience of performing OCB in the organization triggers or blocks the benefits of WFF. Addressing these research questions requires both the traditional methods used for investigating OCB and the work-home facilitation as well as more sophisticated methods. Addressing both questions regarding change vs. stability of the model provides promising research avenues for OCB (Organ, 2018) as well as WFF processes (Williams et al., 2016), though such research is still in its infancy. In line with the recent call, we conducted both a cross-sectional study as well as a daily survey study. The general research model is presented in Figure 1.

Organizational citizenship behavior is defined as “individual behaviors that are discretionary, not directly or explicitly recognized by the formal reward system and promote the effective functioning of the organization” (Organ, 1988, p. 4). Williams and Anderson (1991; derived from Organ, 1988) suggested a parsimonious two-factor conceptualization: *organizational citizenship behavior-individual* (OCBI), comprising behaviors targeted at helping other individuals in the organization; and *organizational citizenship behavior-organizational* (OCBO), which encompasses extra-role behaviors directed toward the organization in general. However, as these dimensions are very highly correlated with OCB, it may also be considered unidimensional (for meta-analyses, see LePine et al., 2002; Hoffman et al., 2007). Thus, the current study adopted this unidimensional approach. In the current work, we suggest that employees who go the extra mile by performing OCB acquire new skills and qualities that can also be applied to family roles.

Work-family facilitation can illustrate this positive perspective (Wayne et al., 2007; Williams et al., 2016), with WFF being defined as the degree to which an employee’s involvement in the work domain boosts functioning in the family domain (Wayne et al., 2004). The resource gain development model (Wayne et al., 2007) suggested that individuals have a natural tendency to achieve, develop, and grow through the highest levels of functioning in their organization as well as in their family system. Thus, employees’ working life can increase the likelihood of their acquiring exposure to new experiences and skills that can also benefit their family life (Frone, 2003). For example, if employees acquire multi-tasking skills in the course of their job responsibilities, these skills may improve their functioning in performing simultaneous behaviors in another social system, such as child-raising. This model can expand our understanding of the impact of OCB on WFF, as OCB can initiate the acquisition of potential gains, which appear to be the starting point for WFF processes. The theory suggests that the workplace provides several potential gains, both personal resources (e.g., self-efficacy, positive affect) and environmental resources (e.g., developmental opportunities, support, job prestige), all contributing to WFF (Wayne et al., 2007). Previous research has suggested that OCB provides employees the opportunity to learn new skills; thus, by helping others solve problems, employees may find that their own problem-solving skills have been enhanced (Roberts and Davenport, 2002). By offering advice, employees take the other’s perspective, which aids in generating new ideas (McGrath et al., 2003). In addition, OCB may contribute to building strong social ties (Levin et al., 2011; Bolino and Grant, 2016), provide opportunities for personal development, and for gaining status and respect (Flynn et al., 2006; Burris, 2012). Moreover, daily performance of OCB increases daily feelings of vigor (Lam et al., 2016) and positive emotions (Glomb et al., 2011), which can be considered as potential resources enabling WFF (Wayne et al., 2007).

Based on the noted arguments, that OCB provides both personal and environmental gains and expands one’s resources, it has been suggested that OCB increases work-family enrichment (WFE) through increased personal skill development among Chinese employees (Kwan and Mao, 2011). The current work focuses on WFF. To highlight the distinction between work-family facilitation and work-family enrichment, the former (WFF) characterizes the benefits of work to the operation of the family system, whereas WFE is seen as more specific, focusing on the gains of the individual. Thus, WFF refers to the way positive participation in the working role makes the fulfillment of family role better or easier (Wayne et al., 2007). Similarly, we hypothesize that the personal and environmental gains of performing OCB would be positively transferred to WFF as well. Hence, we offer the following hypothesis:

H1: OCB is positively associated with WFF

We suggest that the impact of work experiences on WFF may also be a function of the individual’s personality characteristics, a notion backed by several theories. For example, both the general theoretical framework of COR (Hobfoll, 2002; Halbesleben et al., 2014; Hobfoll et al., 2018), as well as the work-home resources model (WH-R; Ten Brummelhuis and Bakker, 2012) recognize that the perception of the workplace experience as a potential resource or loss is tied to one’s personality traits (which are considered key resources). In addition, Bolino et al. (2018) suggested that OCB comprises a meaningful workplace experience that can either free up resources or deplete them. For example, attending meetings and assisting colleagues can either expand one’s personal knowledge and skills or introduce additional burdens such as time consumption and the inability to complete one’s own work assignments. They also acknowledged the need to track the key personality traits that act as potential moderators of OCB and workplace outcomes.

Though theoretically supported, only few attempts have examined these ideas empirically, exploring the impact of personality traits. For example, Halbesleben et al. (2009) suggested that conscientiousness buffered the negative impact of OCB on work-family conflict. Furthermore, extraversion facilitates the impact of OCB on positive emotions (Glomb et al., 2011). Considering that attachment is a grounded theory with applications to many aspects of psychology (e.g., developmental, social, clinical) and impacts organizational outcomes above and beyond Big-5 personality traits (Richards and Schat, 2011), we surmise that it can also be theoretically considered a possible moderator to OCB and the positive benefits of WFF.

Among individual-difference variables, attachment theory can offer a compelling framework and can serve as a potential key resource mechanism. Attachment orientation comprises personality-related characteristics that reflect internal working models of self, others, and one’s interpersonal relationships (Bartholomew and Horowitz, 1991). These orientations are manifest throughout the life span in a variety of ways, such as emotion regulation, handling interpersonal relations, and the capacity to cope with life challenges (for reviews, see Mikulincer and Shaver, 2017, 2019).

According to Bowlby (1982), early interactions between children and their primary caregivers impact the way we connect, interact, and relate to other people, and affect our social world from “candle to grave” (Mikulincer and Shaver, 2017). Children who experience responsive and sensitive care grow to feel safe and secure in the world and in their relationships with others. The sense of attachment security is considered a resource that promotes social and personal adjustments, including managing stress and challenges (Bowlby, 1982). The positive history of interactions with certain attachment figures strengthens a person’s sense that problems can be resolved, obstacles can be overcome, and goals can be attained (Mikulincer and Shaver, 2017).

However, those who experience intensive or inconsistent responsiveness as children may develop defensive perceptions of their self and their interpersonal relationships. Two insecurely attached dimensions have assumed a prominent presence in the attachment literature (Brennan et al., 1998). Individuals higher in attachment *anxiety orientation* are likely to have received inconsistent care during their childhood. To handle unpredictable interactions, they are inclined to adopt an overly dependent strategy as a means of eliciting attention and care from others. In adulthood, individuals high in attachment anxiety, lacking a sense of their own self-worth, tend to worry if others who were thought to be close will be available when needed. Although individuals with higher levels of attachment anxiety orientations tend to feel underappreciated, they still desire to have close relationships.

Individuals with higher levels of *avoidance orientation* are likely to have experienced unresponsive or rejecting interactions with their primary caregiver, leading to detachment or compulsive self-reliance in interpersonal relationships. They tend to distrust the goodwill of their relationship partners and strive to maintain autonomy and emotional distance from them. They report little desire or willingness to emotionally engage with others (Brennan et al., 1998). These individuals strive to maintain self-reliance and tend to downplay their distress and sense of vulnerability (Mikulincer and Shaver, 2017). Furthermore, individuals with higher levels of avoidance orientation prefer to keep others at a distance, tend to distrust others, and express indifference toward other people (Mikulincer, 1998; Reizer et al., 2012, 2013; Mikulincer and Shaver, 2017).

Finally, individuals low in both dimensions are considered to be possessing *secure attachment orientations*. Their successful history of trust in relationships leads them to develop broader social skills (Lopez, 2009). Their positive interpersonal orientation promotes their ability to cope more successfully with external workplace stressors and to solve problems more effectively (for reviews, see Mikulincer and Shaver, 2017; Reizer, 2019). These advantages provide them the personal resources to enjoy the benefits of OCB which, in turn, contribute to higher levels of WFF than can be expected from individuals with higher levels of anxiety and avoidance.

This two-dimensional framework is considered a validated framework for measuring attachment in the organizational domain (Richards and Schat, 2011; Yip et al., 2018). Thus, it has been shown that avoidance and anxiety predict employee dissatisfaction, conflicts, and burnout at the workplace (Harms, 2011; Reizer, 2015). Converse associations characterize individuals with lower levels of anxiety and avoidance orientations—these being the more securely attached individuals. Although much of the attachment literature has focused on the direct impact of attachment orientations, scholars have recently explored the moderating effects of these orientations as having a significant role in various organizational relationships. For example, it has been suggested that attachment anxiety orientation moderates the associations between leadership support and self-efficacy, whereas avoidance orientation moderates leadership support-employee motivation associations (Wu and Parker, 2017). Another study suggested that autonomy and desired organizational outcomes are stronger for individuals with lower levels of attachment avoidance (i.e., more secure ones; Littman-Ovadia et al., 2013). These few attempts led Yip et al. (2018) to encourage organizational researchers to investigate the moderating role of attachment orientations.

Specifically, individuals with higher levels of attachment anxiety tend to have fewer stress management skills. They are inclined to rely on hyper-activating strategies, involving amplifying distress cues and negative feelings, hypersensitive proximity-seeking reactions, self-perception of vulnerability, and more severe emotional and physical symptoms (Hazan and Shaver, 1990; Richards and Schat, 2011). Consequently, they tend to feel distressed and burned out at work (Reizer, 2015) and report frustrations in managing job demands (Harms, 2011). Their feelings of vulnerability and excessive distress make them less likely to derive any personal gain from performing OCB, a behavior that might evoke feelings of distress (Bolino et al., 2018).

Employees with higher levels of attachment avoidance, defensively denying the value and importance of close relationships, are, therefore, less likely to establish friendly relationships with others (Hazan and Shaver, 1990; Richards and Schat, 2011). This reluctance to establish social connections may, in turn, impair their ability to enjoy the potential social gains of OCB. In addition, they are excessively involved in their work (Hazan and Shaver, 1990) and are more concerned about their overtime work hours (Hardy and Barkham, 1994). These tendencies can be assumed to impede their gains from performing OCB, partially because they might refer to it as an unwanted interruption and time consuming rather than as an opportunity.

Finally, individuals who are lower in avoidance and anxiety (i.e., more securely attached) tend to report higher levels of self-efficacy at the workplace, cope more effectively with stressors, and take steps to address them (Hazan and Shaver, 1990; Lopez, 2009). Furthermore, they typically enjoy interpersonal interactions at the workplace (Yip et al., 2018). We assume that these employees would have enough personal resources to accrue potential gains from performing OCB.

In sum, whereas individuals with lower levels of avoidance and anxiety possess more ego resources, allowing them to enjoy the benefits of extra-role activity, individuals with higher levels of avoidance and anxiety might be more wary of the risk of resource *loss* in performing OCB. Given these distinct strategic orientations, individuals who are higher on avoidance orientations are inclined to prioritize their own job requirements and are more likely to appreciate social interactions as time-consuming (Hazan and Shaver, 1990). Thus, they are more likely to decrease their potential benefits from performing OCB. However, individuals with higher levels of anxiety orientations might be more concerned with the stressful distractions stemming from having to perform the extra-role requirements. In light of the dynamics of attachment orientations in the workplace, we offer the following hypotheses:

H2: Attachment orientations will moderate the associations between OCB and WFF.

H2a: OCB will decrease WFF for individuals with higher levels of attachment anxiety orientations than for individuals with lower levels of attachment orientations.

H2b: OCB will decrease WFF for individuals with higher levels of attachment avoidance orientations than for individuals with lower levels of attachment avoidance orientations.

## Study 1

In Study 1, we examined whether OCB would predict WFF and whether attachment orientations moderate this association. For this purpose, we used a cross-sectional design with multiple sources based on a sample of employees in a pharmaceutical company. Participants completed self-report measures of WFF and attachment dimensions, and their direct supervisors assessed employee citizenship behavior.

### Materials and Methods

#### Participants

The sample comprised 90 Israeli employees (30 men; 60 women), all full-time workers in a pharmaceutical company. Findings revealed that about 48% held an academic degree, and 72% reported earning an above-average salary. Their direct supervisors, spread over several departments, participated in the current study and provided employee OCB measures. The employees’ average age was 28.78 (*SD* = 6.90).

#### Measures

##### Work-family facilitation (WFF)

WFF was assessed by using the four-item Work-Family Spillover scale (Wayne et al., 2004), measuring the extent to which the skills, behaviors, or positive mood from work positively influence one’s role in the family (e.g., “The things you do at work help you deal with personal and practical issues at home”). The scale was translated into Hebrew using a bi-directional translation process by two English–Hebrew native speakers, with some modification to enhance the flow of the translations. Participants indicated how often they had experienced the behaviors described in each item during the last week using a five-point Likert-type scale, ranging from 1 (“never*”*) to 5 (“all the time”). For the present sample, the scale yielded a Cronbach alpha of 0.66, comparable to that reported by Wayne et al. (2004).

##### Attachment orientations

Attachment orientations were assessed with the 36-item Experiences in Close Relationships scales (ECR; Brennan et al., 1998). We used the Hebrew version of ECR, translated by Mikulincer and Florian (2000), and previously used by Geller and Bamberger (2009) to assess call center employees. Participants rated the extent to which each item was descriptive of their feelings in close relationships on a seven-point Likert-type scale, ranging from 1 (“not at all”) to 7 (“very much”). Eighteen items assessed attachment anxiety (e.g., “I worry about being abandoned”), and 18 items assessed avoidance (e.g., “I prefer not to show a partner how I feel deep down”). Although Brennan et al. (1998) demonstrated the orthogonality and discriminant validity of the anxiety and avoidance subscales, as reflected in previous studies (e.g., Geller and Bamberger, 2009; Reizer, 2019), the two subscale scores were significantly correlated in the current sample. For the current sample, Cronbach’s αs were 0.91 for the anxiety items and 0.79 for the avoidance items. We further examined the confirmatory factor analysis (CFA) on the attachment scale. A bifactorial model achieved a good fit [χ^2^(6) = 7.284, *p* = 2.95; RMSEA = 0.049, CFI = 0.997, TLI = 0.*9*91, NFI = 0.981] as opposed to the alternative single-factor model [χ^2^(9) = 18.827, *p* = 0.027, RMSEA = 0.11, CFI = 0.973, TLI = 0.*9*56, NFI = 0.951]. The two-factor model demonstrated a significant increase of χ^2^ in comparison with the one-factor model, Δχ^2^(3) = 18.827, *p* < 0.01.

##### Organizational citizenship behavior (OCB)

Lee and Allen’s (2002) 16-item scale was administered to supervisors to assess the 90 employee participants on individual-oriented OCB (OCBI; e.g., “Gives up time to help others who have work or non-work problems”) and organization-oriented OCB (OCBO; e.g., “Defends the organization when other employees criticize it”). This scale was translated into Hebrew and was previously used to evaluate Israeli employees (Reizer et al., 2019). Items were presented on a seven-point Likert-type scale, ranging from 1 (“never”) to 7 (“always”). Both the OCBI and OCBO scales achieved satisfactory internal consistency reliabilities (0.95 and 0.93, respectively). The two OCB scales were highly correlated (*r* = 0.63, *p* < 0.001). Therefore, following, LePine et al.’s (2002) recommendations, we averaged all 16 items for a general construct of OCB. Cronbach’s alpha for this index for the current sample was 0.95.

### Results

#### Confirmatory Factor Analysis

In the first step, a CFA was used to test the measurement model. The model included four latent factors: two independent variables (avoidance and anxiety), OCB, and WFF. Overall, the measurement model achieved a good fit, χ^2^(38) = 45.85, *p* = 0.17, RMSEA = 0.048, CFI = 0.982, TLI = 0.*9*74, NFI = 0.907. Alternative models were examined. For example, the combined model examined three factors (where two attachment styles were combined into one factor), indicating a reasonable model fit, χ^2^(41) = 55.31, *p* = 0.06, RMSEA = 0.063, CFI = 0.96, TLI = 0.*9*5, NFI = 0.88. In addition, an alternative CFI factor combining all items into one factor was also examined, suggesting a poor model fit, χ^2^(44) = 121.06, *p* = 0.000, RMSEA = 0.14, CFI = 0.82, TLI = 0.78, NFI = 0.75. The four-factor model showed a significant increase of χ^2^ in comparison with the one-factor model and the three-factor model, Δχ^2^(6) = 75.21, *p* < 0.001 and Δχ^2^(3) = 9.45, *p* < 0.05, respectively. Means, standard deviations, and correlations between study variables are presented in Table 1. Results indicated that OCB and WFF were significantly correlated.

**TABLE 1:** Means, standard deviations, and zero-order bivariate correlations.

	***M***	***SD***	**1**	**2**	**3**	**4**
(1) WFF	3.00	0.77	(0.65)			
(2) Avoidance orientation	2.79	0.78	0.14	(0.79)		
(3) Anxiety orientation	2.89	1.14	–0.06	0.40^∗∗∗^	(0.91)	
(4) OCB	4.89	1.26	0.22^∗^	–0.01	–0.03	(0.95)

*^∗^*p* < 0.05, ^∗∗∗^*p* < 0.001.*

**FIGURE 2:**
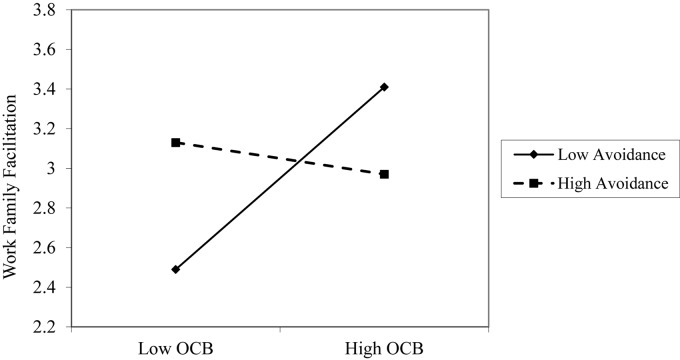
Interaction between avoidance orientation and OCB in predicting WFF in Study 1.

A two-step hierarchical regression analysis predicted WFF. In Step 1, we included OCB scores as well as attachment anxiety and avoidance. In Step 2, we entered two interaction terms: Avoidance X OCB and Anxiety X OCB. All measures were first centered on their sample means. Regression coefficients are presented in Table 2. As indicated in Table 2, multicollinearity was not a concern, as tolerance values were 0.96 and higher while VIF < 1.35 (Lavery et al., 2019). The analysis revealed that higher OCB scores, as reported by the supervisors, predicted higher levels of WFF (β = 0.20, *p* < 0.05), thus supporting H1. Furthermore, findings indicated that attachment avoidance moderates the associations between OCB and WFF (β = −0.23, *p* < 0.05). The observed moderator effect was consistent with our expectations. A simple slope analysis (Aiken and West, 1991) indicated that the relationship between OCB and WFF was stronger for participants having lower levels of attachment avoidance (β = 0.38, *p* < 0.001) than for those high in attachment avoidance (β = −0.05, ns). Figure 2 graphically depicts this interaction. Thus, Hypothesis 2b was supported for attachment avoidance. The moderating role of attachment anxiety on the association between OCB and WFF was non-significant; thus, H2a was not supported.

**TABLE 2:** Standardized regression coefficients predicting WFF from employee attachment orientations and supervisor OCB ratings (Study 1).

	***B***	***SE***	**β**	**Tolerance**	**VIF**
**Step 1**					
OCB	0.19	0.09	0.23^∗^	0.99	1.00
Avoidance orientation	0.15	0.11	0.16	0.79	1.26
Anxiety orientation	0.01	0.09	0.01	0.79	1.26
Δ *R*^2^		0.08^∗^			
**Step 2**					
OCB	0.18	0.07	0.20^∗^	0.98	1.02
Avoidance orientation	0.18	0.11	0.15	0.74	1.35
Anxiety orientation	–0.03	0.09	–0.04	0.77	1.31
Avoidance X OCB	–0.27	0.13	−0.23^∗^	0.80	1.24
Anxiety X OCB	0.01	0.08	0.01	0.85	1.17
Δ *R*^2^		0.05^∗^			
Total *R*^2^		0.13^∗^			
Total F		2.01^∗^			

*^∗^*p* < 0.05.*

To examine the possibility that the direction was in reverse order, with WFF leading to OCB and anxiety and avoidance moderating the association, a second multiple regression analysis was performed. The findings showed that the variance explained by WFF and the interaction term were not significant, *F*(6,88) = 0.95, *p* = 0.46. Therefore, reverse causality was not confirmed.

### Discussion

The results support our contention that OCB scores, as reported by direct supervisors, predict WFF. Our results are consistent with the more recent perspective of *enrichment*, as discussed by Lam et al. (2016). In addition, we suggest that the impact of OCB on WFF needs to consider individual differences before drawing definitive conclusions. In line with expectations, employees low on avoidance are likely to benefit from performing OCB at the workplace in terms of increasing their WFF, whereas individuals high on avoidance are less likely to experience these benefits. In addition, the current results suggested that avoidance and anxiety are significantly correlated. The significant correlations are consistent with a previously published meta-analysis of attachment measures that argued for the association between avoidance and anxiety dimensions while using ECR, especially when examining samples outside of North America that included older and non-university participants (Cameron et al., 2012). Following Cameron et al.’s (2012) recommendation, we statistically addressed shared variance in our analyses by including both dimensions of attachment as predictors in the same step in the regression model and by examining multicollinearity.

Though the findings of Study 1 were quite clear, several limitations should be acknowledged. Firstly, the data do not provide clear evidence for the direction of the effect. The cross-sectional design does not permit determining causality regarding the associations between OCB and WFF. Second, OCB outcome measures were based on supervisor reports; therefore, one can speculate that impression management processes may be more dominant and perhaps colored supervisor OCB reports, impairing their objectivity (Bolino et al., 2018). Supervisor ratings of OCB have been regarded as less vulnerable to social desirability and self-presentation biases relative to self-ratings. However, supervisors can be affected by various factors including halo effects, the rater’s own views and impressions of the employee’s behavior in general rather than OCB in particular and by having observed only a limited number of OCBs (for review and meta-analysis, see Carpenter et al., 2014). As we asked the supervisors to rate their employees’ OCB, it might be more sensitive to these biases. Finally, previous research has demonstrated that both WFF (Butler et al., 2005) and OCB (Lam et al., 2016) are dynamic constructs. A within-person method reduces retrospective bias, measurement error, and biased self-serving attribution (Maertz and Boyar, 2011; Germeys et al., 2019). As such, a diary methodology was applied in Study 2.

## Study 2

Study 2 sought to account for one of the major limitations of Study 1, namely, the potential daily fluctuations of OCB and WFF. To better understand the OCB- WFF effects, it is important to examine both within- and between-person effects, as they address different research questions. Within-person effects focus on short-term changes within an individual and are particularly suited to identify relationships that hold within the person; thus, they are appropriate for examining the linkage between daily fluctuations. In contrast, between-person effects are better suited for addressing the more lasting associations observed among employees, such as the relationships between general expression of OCB and general perception of facilitation. Indeed, the periodic call in the literature for simultaneous consideration of within- and between-person relationships has been strongly recommended to achieve a more complete evaluation of the dynamic nature of OCB (Organ, 2018) and work-family interface (Williams et al., 2016). It has been suggested that daily performance of OCB daily could contribute to daily positive emotions and vigor at the end of the working day (Glomb et al., 2011; Lam et al., 2016). Therefore, the contribution of OCB to WFF can be explained by the daily fluctuation of resources, generated by performing OCB. However, based on Study1’s findings, the following hypotheses are posited:

H3: Daily OCB will enhance daily WFF.

We further examined whether employee’s intra-individual OCB and daily WFF associations are moderated by a more stable personality attachment orientation. More specifically, the work-home resources model (WH-R; Ten Brummelhuis and Bakker, 2012) theoretically recognized that stable personality traits could serve as potential moderators at the between-level of analysis of the daily fluctuation of more transferable workplace experiences on positive work-family interface. Adapting this theoretical framework to the current study, we examined whether the between-person variation in attachment can moderate OCB- WFF fluctuations. Based on Study 1’s findings, we presumed that employees with lower levels of anxious and avoidant orientations are likely to view daily OCB episodes as opportunities for growth and advancement, whereas employees characterized by higher levels of anxiety or avoidance orientations are likely to find these daily OCB episodes more stressful or demanding.

H4: Attachment orientations moderate the associations between daily OCB and daily WFF.

H4a: Daily OCB enhances daily WFF for individuals lower on anxiety orientations than for individuals higher on anxiety orientations.

H4b: Daily OCB enhances daily WFF for individuals lower on avoidance orientations than for individuals higher on avoidance orientations.

### Materials and Methods

#### Participants and Procedure

A total of 138 Israeli employees completed the baseline survey. Each participant received an email describing the purpose and the procedure of the research project and was directed to a link containing a questionnaire with baseline demographic and attachment-orientation items. A few days later, responding participants, all employees in various organizations, were asked if they would be willing to complete a survey after returning home from work for 10 working days. All data were collected online using electronic surveys. Participants completed a daily survey at fixed intervals at their homes following their workday; this procedure was instituted to examine the dependent variable (WFF) in real-time. In addition, participants completed an OCB scale describing their working day activities. As an additional check, the time at which each survey was submitted was examined to ensure that the surveys were being completed at the appropriate time (assuring completion after their workday). Daily responses to the online surveys began on a Sunday (the first workday of the week in Israel). After completing the series of questionnaires, participants were debriefed and thanked. As a small token of appreciation, those completing two working weeks (10 days) of daily surveys were offered the opportunity to enter a single drawing for a $28 bonus, a procedure they had been informed of as an incentive to participate. Winners of four monthly drawings were drawn randomly from all entries collected during the month and were awarded their prizes through email correspondence.

In total, 108 employees (1080 responses) completed all the daily questionnaires (10 answering days for 108 respondents), thus comprising the final study sample. As only partial data were available for 30 of the participants, these were dropped from the analysis. No significant differences were found between the excluded and the final groups in age, *t*(136) = 0.48, *p* = 0.63; gender, χ^2^(1) = 2.59, *p* = 0.11; job tenure, *t*(135) = 0.86, *p* = 0.38; number of weekly work hours, *t*(123) = 0.26, *p* = 0.80; years of education, *t*(130) = 0.03, *p* = 0.98; attachment avoidance orientations, *t*(136) = 0.14, *p* = 0.90; and attachment anxiety, *t*(136) = 0.36, *p* = 0.72. Of the final sample, 60% were female, and 58% reported being married or in a relationship. Mean age of the participants was 32.03 (*SD*_*age*_ = 11.37), average hours worked per week were 40.27 (*SD* = 10.2). Education levels ranged from 10 to 21 years (*M*_*education*_ = 14.3, *SD* = 2.4), with 15.5% working in managerial positions.

#### Measures

##### Daily OCBs

We measured OCB using a scale adapted from Lee and Allen (2002). To keep the survey brief, the original scale was slightly altered by Spence et al. (2011) to measure OCB in a daily context. Specifically, participants were asked, “Please indicate if you performed the activities listed below at work today.” Five items examined OCBI, and five examined OCBO. The scale was translated to Hebrew using a bi-directional translation process carried out by two English-Hebrew native speakers. Sample items included “…willingly gave your time to help others having work-related problems” and “…assist others with their duties.” Consistent with prior research supporting a unidimensional view of citizenship behavior (LePine et al., 2002), interpersonal and organizational citizenship items were summed to form a measure of overall citizenship behavior. Sample items included, “Today, I helped others who needed it,” and “Today, I did things that were not required of me, but that helped the organization.” Cronbach’s α for the current sample, calculated for each of the 10 days, ranged from 0.89 to 0.96.

##### Daily work-family facilitation

Work-family facilitation, as conceptualized by Butler et al. (2005), was adapted to the daily measures. The items mostly tapped affective and cognitive aspects of WFF (e.g., “I had a good day at work today, so I was a happier person when I got home”; “Doing my job gave me a more positive attitude at home today”; and “My mood when I left work made me a better person at home today”). The scale was translated to Hebrew using a bi-directional translation process carried out by two English-Hebrew native speakers. Each item was assessed on a five-point Likert-type scale. Cronbach’s α for this sample, calculated for each of the 10 days, ranged from 0.89 to 0.96.

Time-lagged WFF was created for WFF. These time-lagged measures were calculated by taking each employee’s WFF score from the previous day. This measure was used to control for potential confounding effects, such as the cross-correlation of WFF over the sequence of the 10 repeated working days and to check for stability versus variability in the outcome (Bliese and Ployhart, 2002).

##### Attachment orientations

Attachment anxiety and avoidance were assessed with the 36-item Experiences in Close Relationships scales (ECR; Brennan et al., 1998). Participants rated the extent to which each item was descriptive of their experiences in close relationships on a seven-point Likert-type scale, ranging from 1 (“not at all”) to 7 (“very much”). Eighteen items tapped attachment anxiety (e.g., “I worry about being abandoned”), and 18 items tapped avoidance (e.g., “I prefer not to show a partner how I feel deep down”). Cronbach’s α for this sample was 0.88 for the attachment anxiety orientation items and 0.82 for the avoidance orientation items.

### Results

#### Internal Consistency Reliability

The scales internal reliability was assessed by estimating the level-specific omega coefficients, as single-level estimates of reliability, such as Cronbach alpha coefficients, do not accurately reflect a scale’s actual reliability when variance exists at multiple levels (i.e., within- and between-person variance; Geldhof et al., 2013). The internal between-person reliabilities of the scale for OCB (omega = 0.80) and WFF (omega = 0.94) for the current sample were satisfactory.

#### Confirmatory Factor Analysis

A multilevel CFA was performed, in which we specified WFF and OCB at the within-person and between-person level, with attachment only specified at the between-person level. Overall, our bifactor model achieved a good to reasonable fit at the within-level of analysis [χ^2^(64) = 564.09, *p* = 0.00, RMSEA = 0.07, CFI = 0.92, TLI = 0.90, SRMR = 0.06]. Each item loaded significantly, and in the expected direction, onto its respective latent factor. This model fit the data better than the alternative model, which includes a single general factor [χ^2^(65) = 2089.98, *p* = 0.00, RMSEA = 0.15, CFI = 0.66, TLI = 0.59, SRMR = 0.15]. The bifactor model showed a significant increase of χ^2^ as opposed to the single-factor model [Δχ^2^(1) = 1525.89, *p* < 0.001]. In order to demonstrate the empirical distinction between anxiety and avoidance dimensions, we conducted a CFA at the between-person level and demonstrated that a two-factor model [χ^2^(7) = 13.89, *p* = 0.05, RMSEA = 0.09, CFI = 0.98, TLI = 0.96, NFI = 0.97], in which items load onto their corresponding latent factor, fit the data better than the alternative model, which includes a single general factor of attachment orientation [χ^2^(8) = 107.18, *p* = 0.00, RMSEA = 0.34, CFI = 0.75, TLI = 0.52, NFI = 0.74]. The bifactor model showed a better model fit as opposed to the single-factor model [Δχ^2^(1) = 93.29, *p* < 0.001]. Table 3 presents the aggregated correlation matrix. As seen in Table 3, OCB and WFF were significantly correlated, thus supporting H1.

**TABLE 3:** Means, standard deviations, and Zero-Order bivariate correlations (Study 2).

	***M***	***SD***	**OCB**	**WFF**	**Anxiety**
OCB	4.73	2.22			
WFF	3.05	0.82	0.35^∗∗∗^		
Anxiety orientation	2.84	1.02	–0.04	0.06	
Avoidance orientation	3.21	0.88	0.06	–0.04	0.33^∗∗∗^

**^∗∗∗^p* < 0.001.*

#### Multilevel Analyses

To examine the effect of OCB over WFF on the daily level and person level, we used a two-level hierarchical model, in which daily measures were nested within each person (Bryk and Raudenbush, 1992). This analysis controls for dependencies in an individual’s report across multiple days. For Level 1, regarding the intra-individual domain, we included the effects of the time-varying predictors (i.e., OCB and WFF). Variables at Level 1 for the daily domain were centered on the person mean across the 10 working days of the study. Variables at Level 2 for the interpersonal level, such as attachment orientations, were grand-mean centered.

The models were calculated (Bryk and Raudenbush, 1992) using HLM 6.1 software, in which OCB and WFF were the Level 1 units of analysis, and attachment orientations were the Level 2 units. Before conducting the analyses of daily relationships between OCB and WFF, we first investigated the amount of variance in the study variables attributable to within-person and between-person sources. Therefore, we first calculated the means and the percentages of variance of the within and the between levels (Nezlek, 2008; Kafetsios, 2019). Findings indicated that a substantial proportion of the variance in these variables could be attributed to within-person differences, supporting a multilevel approach (Marcoulides and Schumacker, 2009). The results are presented in Table 4. To control for between-person effects, the daily predictor scores were cluster-mean centered. In other words, the previous day’s outcome scores represented a participant’s deviation from his or her mean score on the variable across the days that the individual completed the diary entries. Furthermore, to rule out daily serial dependency, we controlled for the previous day’s outcome. Thus, in predicting the current day’s WFF, the previous day’s WFF was partialed out. To test the intra-individual and cross-levels hypotheses, we used hierarchical linear modeling (HLM5; Bryk et al., 2000). In order to assess the influence participants’ OCB had on their WFF at Level 1, as well as the moderating role of attachment at Level 2, we used the following model. Level 1:

y⁢i⁢j=b⁢0⁢j+b⁢1⁢j⁢(O⁢C⁢B)+b⁢2⁢j⁢(W⁢F⁢F⁢d⁢a⁢y⁢i-1)+r⁢i⁢j

Level 2:

β⁢0⁢j=γ⁢00+γ⁢01⁢(A⁢n⁢x⁢i⁢e⁢t⁢y)+γ⁢02⁢(A⁢v⁢o⁢i⁢d⁢a⁢n⁢c⁢e)+u⁢0⁢j

β⁢1⁢j=γ⁢10+γ⁢11⁢(A⁢n⁢x⁢i⁢e⁢t⁢y)+γ⁢12⁢(A⁢v⁢o⁢i⁢d⁢a⁢n⁢c⁢e)+u⁢1⁢j

β⁢2⁢j=γ⁢20+γ⁢21⁢(A⁢n⁢x⁢i⁢e⁢t⁢y)+γ⁢22⁢(A⁢v⁢o⁢i⁢d⁢a⁢n⁢c⁢e)+u⁢2⁢j

**TABLE 4:** Multilevel summary statistics.

	**Mean**	**ICC**	**Variance**	
			
		**% of variance within persons**	**Between-person**	**Within- person**
WFF	3.05	0.49	0.58	0.61
OCB	4.70	0.61	4.21	2.71

Table 5 presents the results of the HLM output for predicting WFF. The results included the main effects of OCB, the main effects of anxiety and avoidance orientations, and the interactions between attachment orientations and OCB at the second level of analysis (between individuals). Consistent with H3, results for the daily domain (Level 1) revealed that after controlling for previous day WFF, daily OCB was a significant predictor of greater daily WFF. In other words, participants’ reports of greater daily OCB at work predicted a concomitant rise in WFF, thus supporting H3.

**TABLE 5:** Daily OCB predicting daily WFF (Study 2).

**Effect**	**Coefficient**	***SE***
**Daily level**		
Previous day WFF	0.10^∗∗∗^	0.03
OCB	0.07^∗∗∗^	0.01
**Person level**		
Avoidance orientation	–0.04	0.10
Anxiety orientation	–0.04	0.08
Cross-level interactions		
Avoidance ^∗^ OCB	–0.05^∗∗^	0.01
Anxiety ^∗^ OCB	0.02	0.01

*^∗∗^*p* < 0.01; ^∗∗∗^*p* < 0.001.*

**FIGURE 3:**
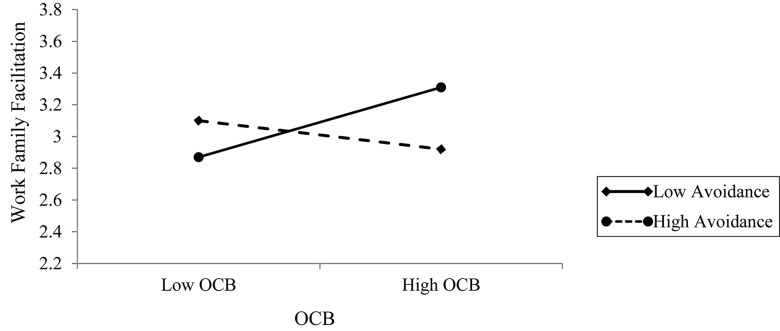
Interaction between avoidance orientation and daily OCB in predicting daily WFF.

A significant two-way interaction between OCB and attachment avoidance (Level 2) was observed, providing support for H4b. An examination using Preacher et al.’s (2006) procedure indicated that OCB predicted greater WFF for individuals with low avoidance scores (−1 *SD*; Ɣ = 0.05, *p* < 0.05), whereas OCB was negatively associated with WFF for individuals with high avoidance scores (+1 *SD*; Ɣ = −0.02, *p* < 0.05). To further probe the interactions and based on previous work, we calculated predicted values for employees who are lower and higher in attachment avoidance orientation as well as lower and higher in performing OCB (Table 6). As can be seen in Figure 3, which graphically depicts this interaction, for employees with lower levels of avoidance orientation, higher OCB was associated with higher WFF. However, at higher levels of avoidance attachment orientation, higher OCB performance was associated with lower WFF, thus supporting H4b. However, the moderating role of anxiety on the association between OCB and WFF (H4a) was not supported.

**TABLE 6:** Predicted values in WFF outcomes for a combination of low and high avoidance orientation and OCB.

		**Avoidance Orientation**	
		**Low**	**High**
**OCB**	Low	2.87	3.1
	High	3.31	2.92

To provide support for the direction of the effect between OCB and WFF, HLM was applied to examine the effect of the previous day’s measure on the next day’s measure of the alternate variable. Findings revealed that the previous day’s OCB was significantly associated with the next day’s WFF (Ɣ = 0.04, *p* < 0.05), whereas the previous day’s WFF was not a significant predictor of the next day’s OCB.

### Discussion

Supporting H1, and in line with Study 1, the diary findings indicate that daily performance of OCB may predict daily fluctuations of WFF, even when controlling for the previous day’s facilitation. Furthermore, in line with our research hypotheses, individuals with lower levels of avoidance orientations tended to benefit from performing OCB practices; they reported that engaging in OCB during working hours contributed to WFF. These employees tend to establish positive and trustful relationships at work (Harms et al., 2016) and enjoy meetings and social events (Hazan and Shaver, 1990), thus facilitating benefits derived from performing extra-role practices. As for employees with higher levels of attachment avoidance orientations, their daily experience with OCB during working hours impaired their WFF. They tend to perceive personal relationships as burdensome and as a depletion of resources (Mikulincer and Shaver, 2017); thus, the daily experience of OCB may be associated with lower WFF levels.

Finally, as noted, anxiety did not moderate OCB and WFF associations. Individuals with higher levels of attachment anxiety find it difficult to handle work overload and job demands (Harms, 2011). However, their longing and need to feel accepted, to belong, and to collaborate at the workplace (Rom and Mikulincer, 2003; Yip et al., 2018) may account for some of the benefits they accrue in performing OCB and explain the inconsistent findings regarding the effects of attachment anxiety orientations at the workplace.

## General Discussion

Our study contributes to the ongoing debate concerning whether OCB is beneficial or harmful to the employees who engage in these activities. Furthermore, the current findings expand our knowledge regarding the need to examine the moderating role of personality types in explaining the OCB-work-family interface (Halbesleben et al., 2009). Specifically, the research findings of both current studies indicate that individuals having lower levels of avoidance orientations derive greater benefit from performing OCB than do individuals with higher levels of avoidance orientations. These individuals seem to have a greater capacity to enjoy interpersonal interactions and are inclined to welcome challenging situations (Mikulincer and Shaver, 2017). Although only a few studies have investigated the moderating role of avoidant orientations (Littman-Ovadia et al., 2013; Dahling and Librizzi, 2015), their findings are consistent with the current findings. In both cases, they reported that individuals with lower levels of avoidance orientations gain more from desired organizational outcomes.

As for individuals with higher levels of attachment anxiety, both current studies indicated that anxiety does not moderate OCB and WFF associations. On the one hand, individuals with higher levels of anxious orientation tend to report heightened responsiveness to negative stimuli and stressors at the workplace (Hazan and Shaver, 1990; Hardy and Barkham, 1994). However, one should consider the crucial importance that individuals with higher levels of anxious orientations give to social interactions and their intense involvement in relationships, both in and outside work (Harms, 2011). In addition, individuals with higher levels of attachment anxiety orientation might be motivated to help others (Reizer and Mikulincer, 2007), even though their efforts may be less effective (Reizer and Mikulincer, 2007; Reizer et al., 2012). These contradicting tendencies explain their ambivalent and, at times, unpredictable behavior at the workplace (see Harms et al., 2016; Wu and Parker, 2017).

We found non-significant associations for both avoidance and anxiety orientations in predicting OCB. These findings are not in line with previous work, which suggested that attachment orientations can predict prosocial behaviors such as OCB (e.g., Little et al., 2011; Richards and Schat, 2011; Reizer, 2019). We assume that the lack of significant associations between OCB and attachment orientations in the current study may stem from several causes, both theoretical and methodological. From a theoretical perspective, while previous studies referred to OCB as a prosocial and a more voluntary activity (e.g., Little et al., 2011; Richards and Schat, 2011), OCB may stem from self-interest motives (such as impression management) and not necessarily from purely altruistic ones (Rioux and Penner, 2001). Furthermore, employees are sometimes pressed and obligated to perform OCB (Bolino et al., 2010). As both researchers and practitioners have recognized the essential role of OCB in employee performance evaluation processes (e.g., Allen and Rush, 1998), individuals with higher levels of anxious and avoidance orientations are also among those requested to perform OCBs, whether or not it suits them. Future work can clarify the underlying conditions where attachment- OCB associations might exist and address the citizenship pressure as a potential moderating mechanism for these associations (or the lack of them).

The lack of significant associations can also stem from methodological issues. In both studies, we sought to decrease common-method variance (CMV; Podsakoff et al., 2012) and self-presentation bias, either by using supervisor reports or by creating a diary design with daily constructive reports. However, correlations between different employees’ self-reported measures and OCB are weak when OCB is assessed by supervisor ratings (Carpenter et al., 2014). Furthermore, within-subject designs might generate small effect sizes, as they are more likely to control for between-person confounding effects, such as personality and general response bias (Charness et al., 2012).

Finally, due to their unique relational perspective, a growing interest has emerged regarding the role of attachment orientations in the organizational domain, and it is highly recommended to examine the moderating role of the attachment construct in shaping individuals and affecting organizational outcomes (Harms, 2011; Yip et al., 2018). Our findings support the notion that attachment personality orientations can serve as an ego resource mechanism and that individuals with lower levels of anxiety and avoidance orientations can gain more from workplace experiences such as OCB. We suggest that attachment orientations can enrich not only our understanding of the effects of daily OCB on work-family processes but can help broaden an appreciation of other aspects of an employee’s life outside of work.

The present study has some limitations that need to be acknowledged. First, caution is warranted in drawing conclusions regarding causality, as a true experimental design was not used. A longitudinal design encompassing a more extensive period and examining employees during different life stages (e.g., early career, getting married, transition to parenthood) may provide a better and clearer answer to the positive influence of OCB on WFF. Second, the findings explained a relatively small amount of variance in the outcome variables. Nevertheless, the reported effect sizes are comparable to other diary studies (Bakker and Xanthopoulou, 2009; Mojza et al., 2010) as well as to other cross-sectional investigations of OCB and the work-family interface (Carlson et al., 2013). In addition, Zhang et al. (2018), in their meta-analysis, showed that within-domain effects were stronger than the cross-domain effects, results consistent with the present findings. Third, the current investigation adopted the recognized two-dimensional approach (Richards and Schat, 2011, as noted), which does not posit specific hypotheses concerning the securely attached individuals falling at the low end of each of these two dimensions. Future studies may also explore the specific aspects of OCB that explain the predictive effect of OCB over WFF, such as helping, knowledge sharing, and attending workplace meetings. Finally, findings revealed a significant positive correlation between anxiety and avoidance. The significant correlation between attachment orientations was previously found among previous studies using ERC scale (Cameron et al., 2012; Tziner et al., 2014; Reizer, 2019). From a theoretical perspective, these correlations may raise some questions regarding the orthogonal dimensions of attachment. While some researchers assumed anxiety and avoidance to be orthogonal, others have posited that it is not a requirement (for a review, see Cameron et al., 2012). Indeed, even Bowlby (1973), in his original conceptualization of attachment, argued that attachment orientations could be orthogonal in theory but related in practice. In addition, from a methodological perspective, adult attachment literature clearly indicates that the ECR scale is a valid and commonly used measurement of attachment (Mikulincer and Shaver, 2017). However, the ECR anxiety scale includes only one reverse-scored item. This item design might create a response bias, as ECR subscales are expanded at their secure ends in similar ways, possibly resulting in artificial associations (Frías et al., 2015). Future researchers may wish to develop an ECR version to be targeted by self-report measures of attachment at the workplace, a step that might overcome some of the ECR’s limitations.

Theoretically, our findings add to the growing body of research examining the positive effects of OCB (Glomb et al., 2011; Lam et al., 2016). Over the past two decades, scientific attention has acknowledged the advantages of helping others, recognizing that when it comes to mental and physical health, providing support to others can often be very beneficial. Empirically, our study supports the idea that by engaging in behaviors that are social in nature, such as helping another person, you can also help yourself (Doré et al., 2017). We contend that our work complements this previous work and contributes to the current dialogue regarding the positive impact of OCB on the individual’s quality of life. Moreover, OCB carries more positive implications for those lower on avoidance. The moderating role of attachment on the association between OCB and work-family interface can encourage other researchers to address and combine interdisciplinary concepts from clinical, social, and personality psychology perspectives in the work domain and achieve a greater understanding of the role of personality and interpersonal schemas at the workplace.

### Practical Contribution

From a more practical perspective, organizations today are increasingly likely to encourage their employees to work harder, put in longer hours, and be more accessible (Major et al., 2002; Brett and Stroh, 2003). Furthermore, supervisors often consider OCBs in their performance appraisals. Accordingly, employees may feel pressured to engage in high levels of OCBs as a result of their image management at work in order to be seen as cooperative, committed employees (Bolino et al., 2004). Indeed, escalation of OCB may yield some positive consequences, such as gaining psychological, social, and learning benefits (Bolino and Grant, 2016). Some researchers have even concluded that managers could use OCB as a possible managerial mechanism for promoting well-being at the workplace (Glomb et al., 2011). However, we suggest that supervision and training for OCB can help employees attain more benefits from performing OCB than those limited to the workplace by transferring their gains to non-work domains. Increasing consideration of the individual-difference variables—other than cognitive ability—in understanding organizational performance and behavior may significantly improve leadership research and practice (Wu and Parker, 2017).

Supervisors should bear in mind that not all employees may appreciate the relevance of these benefits to them. Individuals with higher levels of attachment avoidance, who tend to be independent and find interpersonal relationships challenging, may view OCB experiences as being associated with potentially negative outcomes. Thus, organizational interventions aimed at increasing WFF may succeed to the extent that they take individual differences into account.

## Conclusion

In carrying out a study based on different data sources (supervisor and employee) as well as a diary study, our work expands the research exploring the benefits of OCB by suggesting that OCB can contribute to WFF. These diverse methods are in line with the need to conduct studies that facilitate a deeper understanding of OCB and WFF over time rather than limit the research focus to detecting discrete episodes of both (Williams et al., 2016). Finally, although the current research provides additional evidence for the commonly accepted notion in social psychology that we can benefit from *doing good* (Carlson and Miller, 1987; Sonnentag and Grant, 2012), individual differences may very well-moderate this effect.

## Data Availability Statement

The datasets generated for this study are available on request to the corresponding author.

## Ethics Statement

The studies involving human participants were reviewed and approved by Prof. Meni Ben Ezra, Dr. Shiri Lavy, and Prof. Michael Dolgin. Written informed consent for participation was not required for this study in accordance with the national legislation and the institutional requirements.

## Author Contributions

AR planned the study and wrote the manuscript. MK contributed to the interpretation of the data and writing of the manuscript. BF contributed to the acquisition of the data and literature search.

## Conflict of Interest

The authors declare that the research was conducted in the absence of any commercial or financial relationships that could be construed as a potential conflict of interest.
